# A comprehensive analysis of the incidence of chromosomal abnormalities in hematologic malignancies post-COVID-19 outbreak in King Chulalongkorn Memorial Hospital, Thailand

**DOI:** 10.2478/abm-2026-0022

**Published:** 2026-06-30

**Authors:** Montakarn Tansatit, Nutcharee Jongpornchai, Suwannee Songchart, Kittipornpan Krajokpap, Hudadini Da-oh

**Affiliations:** Cytogenetic laboratory, Center for Medical Diagnostic Laboratories, Faculty of Medicine, Chulalongkorn University, Bangkok 10330, Thailand; Department of Anatomy, Faculty of Medicine, Chulalongkorn University, Bangkok 10330, Thailand

**Keywords:** chromosome abnormalities, COVID-19 outbreak, cytogenetics, hematologic malignancy

## Abstract

**Background:**

Hematologic malignancies, including leukemia, lymphoma, and bone marrow disorders, are complex cancers often associated with chromosomal abnormalities. The emergence of COVID-19 raised concerns about its potential to induce genomic instability, influencing the prevalence and cytogenetics of these malignancies.

**Objectives:**

This study evaluates whether the COVID-19 pandemic affected the incidence, demographics, or chromosomal abnormalities in hematologic malignancies at King Chulalongkorn Memorial Hospital, Thailand, between 2018 and 2024.

**Methods:**

A retrospective analysis was conducted on 3,077 bone marrow samples processed for cytogenetic evaluation. Samples were categorized into pre-COVID-19 (2018–2019) and post-COVID-19 (2020–2024) periods. Trends in disease incidence, patient demographics, and chromosomal abnormalities were analyzed statistically.

**Results:**

The overall incidence rates of hematologic malignancies, including leukemia, lymphoma, and bone marrow disorders, remained stable throughout the study. Male-to-female ratios and average ages of onset showed no significant changes. Except for a transient rise in chromosomal abnormalities in chronic lymphocytic leukemia during 2023, no new or recurring abnormalities were identified. The mean age of onset for various malignancy categories remained consistent across the study period, and the pandemic did not significantly alter hematologic cancer trends.

**Conclusions:**

The findings indicate that the COVID-19 pandemic did not substantially affect the incidence, cytogenetics, or demographics of hematologic malignancies. These results provide reassurance about the resilience of diagnostic and monitoring systems during the pandemic. Further studies are recommended to explore potential long-term implications of COVID-19 on hematologic malignancies.

Recent studies indicate that COVID-19 infection may contribute to genomic instability in hematopoietic cells, potentially elevating the risk of hematologic malignancies. SARS-CoV-2 has been demonstrated to impact multiple essential processes related to DNA damage repair, cell cycle regulation, and the immune response [1]. Viral proteins can engage with host proteins, including DNA polymerases and centrosomal components, resulting in genomic instability and heightened susceptibility to malignant changes. This effect may be associated with the virus’s capacity to modify the DNA damage response and affect oncogenic pathways such as PI3K/AKT/mTOR and MAPK, which are essential for cellular survival and proliferation [2, 3].

Moreover, research has indicated epigenetic remodeling of hematopoietic stem and progenitor cells (HSPCs) subsequent to severe COVID-19 [4]. These alterations may result in skewed differentiation, favoring myelopoiesis (the generation of myeloid cells) and fostering chronic inflammation, a recognized condition conducive to cancer promotion [5]. Significantly, these epigenetic modifications have been detected in both HSPCs and their differentiated descendants, potentially resulting in prolonged immunological dysregulation and an increased susceptibility to hematologic cancers.

The precise clinical ramifications of these findings remain under examination; however, the increasing body of evidence underscores a concerning association between COVID-19 and cancer pathways [6], necessitating additional research into the virus’s potential role in the development of hematologic malignancies over time.

Hematologic malignancies, such as leukemia, lymphoma, and bone marrow disorders, are intricate diseases marked by clonal expansions of aberrant blood cells, sometimes linked to distinct chromosomal abnormalities. The cytogenetic profile of these disorders is essential for their diagnosis, prognosis, and treatment. Recurrent chromosomal abnormalities, including translocations, deletions, and trisomies, have been recognized as significant indicators in diseases such as acute myeloid leukemia (AML), chronic lymphocytic leukemia (CLL), and multiple myeloma (MM), affecting clinical outcomes and treatment responses.

The emergence of the COVID-19 pandemic presented unparalleled hurdles to global healthcare systems, prompting inquiries on the virus’s possible effects on numerous diseases, including cancers. Although extensive research has examined the impact of SARS-CoV-2 infection on the immune system and its contribution to the aggravation of preexisting diseases [7], there is limited understanding of its capacity to produce or affect chromosomal abnormalities in individuals with hematologic malignancies. Recent evidence indicates that viral infections, including coronaviruses, may induce genomic instability, perhaps resulting in novel carcinogenic occurrences.

Considering the pervasive impact of the COVID-19 pandemic and its significant biological consequences, it is essential to investigate whether SARS-CoV-2 infection has influenced the prevalence or cytogenetic characteristics of hematologic malignancies. This study seeks to assess trends in the prevalence of leukemia, lymphoma, and bone marrow disorders from a cytogenetic viewpoint, concentrating on whether the COVID-19 pandemic resulted in alterations in the frequency or characteristics of recurring chromosomal abnormalities. Through the examination of these trends, we seek to elucidate the possible influence of COVID-19 on the genomic landscape of hematologic malignancies.

Cytogenetic analysis is essential for the diagnosis, prognosis, and treatment planning of hematologic malignancies. It entails the analysis of chromosomal abnormalities in cancer cells, which aids in the identification of specific genetic changes that contribute to the progression of the disease [8]. The precision of these assessments is being further improved by advancements in molecular cytogenetics, including fluorescence *in situ* hybridization (FISH) and next-generation sequencing (NGS) [9].

The central hypothesis of this study is that the stress caused by the COVID-19 pandemic, which includes the viral infection and the subsequent inflammatory aftermath, may have contributed to the emergence or acceleration of chromosomal abnormalities, particularly in hematologic malignancies such as leukemia, lymphoma, and bone marrow diseases. The pandemic has established a distinctive environment that is characterized by increased psychological stress, chronic inflammation, and direct viral effects on the immune system. These factors have the potential to disrupt genomic stability. This disruption may be observed as new or accelerated chromosomal abnormalities, which can occur through the direct damage of DNA or the weakened body’s capacity to preserve genetic integrity. The long-term impact of the pandemic on cancer pathogenesis could be better understood by examining the connections between these stressors and cytogenetic alterations.

## Methods

This study was performed in line with the principles of the Declaration of Helsinki. Approval was granted by the ethics committee of the Faculty of Medicine, Chulalongkorn University (COA no. 1405/2025). Informed consent was obtained from all patients at the time of sample collection for cytogenetic analysis.

This retrospective study examined bone marrow samples submitted to the cytogenetics laboratory at the Center for Medical Diagnostic Laboratories, Faculty of Medicine, Chulalongkorn University between January 1, 2018 and October 31, 2024. The study cohort comprised individuals with suspected or confirmed hematologic diseases, including leukemia, lymphomas, and different bone marrow conditions. The samples were submitted for cytogenetic analysis to evaluate chromosomal abnormalities as a component of the diagnostic assessment.

### Inclusion and exclusion criteria

Patients with karyotype results of onco-hematological diseases as defined by the WHO classification criteria for hematological and lymphoid neoplasms based on the morphology and specific cytogenetic alterations of each malignancy were included in this study [7, 8]. Patients submitted to bone marrow transplantation, patients without the hypothesis of neoplasm diagnosis, and patients presenting symptoms or characteristics associated with neoplasms but without reference to a specific pathology were excluded from the study. Moreover, patients under chemotherapy at the first collection of oncological material and cytogenetic analysis were excluded.

### Data collection

Demographic and clinical information, encompassing age, sex, clinical diagnosis, and cytogenetic findings, was extracted from the laboratory reports. The data were categorized into 2 primary eras for analysis: Pre-COVID-19 Era (January 1, 2018–December 31, 2019) and Post-COVID-19 Era (January 1, 2020–October 31, 2024). The objective was to assess trends in the incidence of hematologic illnesses, the average age of onset, sex distribution, and recurrent chromosomal abnormalities during the COVID-19 pandemic in comparison to the pre-pandemic period.

### Cytogenetic examination

Bone marrow specimens were processed in accordance with established cytogenetic methods. Cultures were developed and utilized for G-banded karyotyping. A minimum of 20 meta-phases were examined each sample, unless aberrant clones were detected, in which case additional metaphase cells were assessed. Karyotypes were delineated in accordance with the International System for Human Cytogenomic Nomenclature (ISCN, 2020). FISH or alternative molecular procedures were conducted when clinically warranted.

Cytogenetic data for each sample were classified as:

– Normal Karyotype: Absence of identifiable chromosomal anomalies.
– Abnormal Karyotype: Chromosomal anomalies characterized by structural or numerical alterations, such as translocations, deletions, amplifications, or complex karyotypes.

### Statistical analysis

Descriptive statistics were used to summarize demographic variables and the distribution of hematologic malignancy subtypes. The number of cases for each disease was compared between the pre-COVID-19 (2018–2019) and post-COVID-19 (2020–2024) periods using the Chi-square test of independence. Fisher’s exact test was applied when expected cell counts were <5. Odds ratios (OR) with 95% confidence intervals (CI) were calculated from 2 × 2 contingency tables to assess changes in disease frequencies across the 2 time periods. Differences in mean age between the pre- and post-COVID groups were analyzed using independent samples *t* tests, with Welch’s correction applied when appropriate. All statistical analyses were performed using IBM SPSS Statistics for Windows, version 27.0 (IBM Corp., Armonk, NY, USA). A *P* < 0.05 was considered statistically significant.

## Results

**Figure 1.**
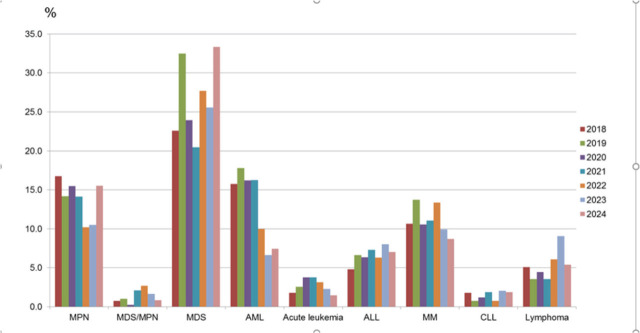
Diagram of distribution by type of disease and by year of the samples processed in the Cytogenetic laboratory during 2018–2024. AML, acute myeloid leukemia; CLL, chronic lymphocytic leukemia; MM, multiple myeloma; MPN, myeloproliferative neoplasms.

A total of 3,077 cases were analyzed, including 1,571 males and 1,506 females, as shown in **Table 1**. The analysis of all data indicated that the incidence proportions of each type of hematological malignancy displayed no observable trend of change. Only the MDS group has demonstrated a slight increase in proportion during the past 3 years. Conversely, specific categories, such as those with AML, demonstrate a propensity for a proportional decrease (**Figure 1**).

**Table 1.** Number of bone marrow samples received by Cytogenetic laboratory during 2018–2024

**Year**	**Total bone marrow samples**	**Total cases included in the report**	**Male**	**Female**
2018	622	394	212	182
2019	704	452	231	221
2020	623	426	226	200
2021	495	425	218	207
2022	601	412	209	203
2023	730	485	258	227
January 1–October 31, 2024	791	483	217	266

Total	4,566	3,077	1,571	1,506

**Figure 2.**
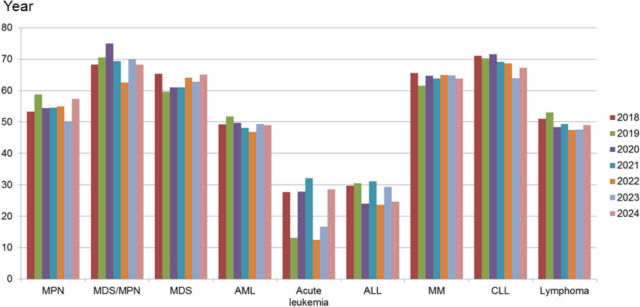
Mean age of onset of patients with hematologic diseases received in the Cytogenetic laboratory for karyotype studies. AML, acute myeloid leukemia; CLL, chronic lymphocytic leukemia; MM, multiple myeloma; MPN, myeloproliferative neoplasms.

The mean age of onset for each category remained stable from 2018 to 2024, showing no trend of variation in patient age (**Figure 2**). As shown in **Table 2**, the comparison of mean patient age across all hematologic malignancy groups revealed no significant differences between the pre-COVID-19 and post-COVID-19 periods (*P* > 0.05 for all groups). These findings suggest that the pandemic did not alter the age distribution at presentation for these malignancies.

**Table 2.** Comparison of mean age of hematologic malignancy patients pre-COVID-19 and post-COVID-19 pandemic

**Disease type**	**Pre-COVID**		**Post-COVID**		*P*
	
	**Mean ± SD**	**n**	**Mean ± SD**	**n**	
MPN	54.6 ± 16.9	130	54.2 ± 19.8	294	0.984
MDS/MPN overlap syndromes	69.4 ± 8.9	7	69.0 ± 16.3	33	0.970
MDS	62.5 ± 24.3	217	62.8 ± 22.8	588	0.988
AML	50.5 ± 17.8	132	48.6 ± 21.4	247	0.914
Acute leukemia (unclassified)	20.4 ± 20.3	17	23.5 ± 26.3	63	0.879
ALL	30.1 ± 23.0	45	26.6 ± 18.4	157	0.868
MM	63.6 ± 13.5	96	64.4 ± 16.0	237	0.949
CLL	70.7 ± 10.6	10	68.1 ± 25.1	35	0.858
Lymphoma	52.0 ± 20.5	34	48.3 ± 25.5	129	0.859

AML, acute myeloid leukemia; CLL, chronic lymphocytic leukemia; MM, multiple myeloma; MPN, myeloproliferative neoplasms.

The average age of patients in the MPN group was between 50 and 60 years, in the MDS/MPM group between 65 and 70 years, in the MDS group around 60 years, in the AML group approximately 50 years, and in the acute leukemia group between 15 and 30 years. The mean age of patients in the ALL group was 25–30 years, in the MM group was around 61 years, in the CLL group was roughly 65 years, and in the lymphoma group was about 50 years (**Figure 2**). OR and 95% CI indicated a statistically significant reduction in the odds of being diagnosed with AML (OR = 0.65, 95% CI: 0.52–0.80) and MPN (OR = 0.79, 95% CI: 0.64–0.98) during the post-COVID-19 period. No significant changes were observed for other disease groups. Fisher’s exact test confirmed a statistically significant decrease in the odds of being diagnosed with AML (*P* = 0.00014) and MPN (*P* = 0.0339) during the post-COVID-19 period. No significant differences were observed for the remaining disease groups (**Table 3**).

**Table 3.** Comparison of hematologic disease frequencies pre- and post-COVID-19 pandemic using OR and Fisher’s exact test

**Disease type**	**OR**	**95% CI**	**Fisher’s *P*-value**
MPN	0.79	0.64–0.98	0.03389
MDS/MPN	1.68	0.74–3.81	0.27847
MDS	0.96	0.81–1.13	0.63511
AML	0.65	0.52–0.80	0.00014
Acute leukemia	1.32	0.77–2.27	0.37042
ALL	1.25	0.89–1.75	0.22189
MM	0.87	0.68–1.11	0.27557
CLL	1.25	0.62–2.52	0.61306
Lymphoma	1.36	0.93–1.99	0.12476

AML, acute myeloid leukemia; CI, confidence interval; CLL, chronic lymphocytic leukemia; MM, multiple myeloma; MPN, myeloproliferative neoplasms; OR, odds ratio.

**Figure 3.**
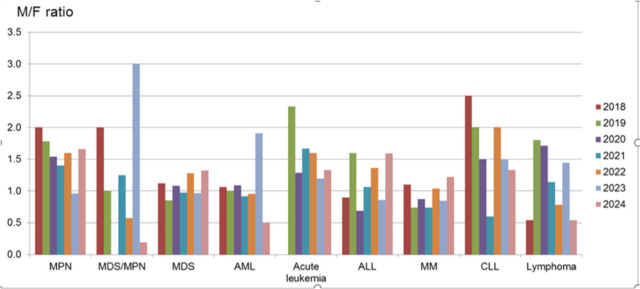
Male-to-female ratio distribution by type of disease and by year of the samples processed in the Cytogenetic laboratory during 2018–2024. AML, acute myeloid leukemia; CLL, chronic lymphocytic leukemia; MM, multiple myeloma; MPN, myeloproliferative neoplasms.

The ratio of male to female patients displayed no observable trend of increase or decrease. In most categories, the incidence among males and females was approximately equal, except for the MPN, CLL, and Lymphoma categories, which were more prevalent in males (**Figure 3**).

**Figure 4.**
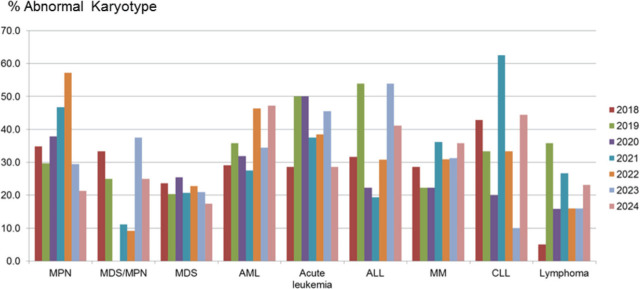
Distribution of chromosomal alterations in hematologic diseases received from 2018 to 2024. AML, acute myeloid leukemia; CLL, chronic lymphocytic leukemia; MM, multiple myeloma; MPN, myeloproliferative neoplasms.

Statistical comparison of the percentage of chromosomal abnormalities before and after the COVID-19 pandemic revealed no significant differences across all disease categories (*P* > 0.05 for all comparisons). Proportion tests confirmed that the prevalence of chromosomal abnormalities remained statistically stable for MPN, MDS/MPN, MDS, AML, and acute leukemia between the pre-COVID-19 (2018–2019) and post-COVID-19 (2020–2024) periods. Cytogenetic findings revealed no significant increase in chromosomal aberrations across any hematological cancer group, except in 2023, when patients with CLL demonstrated a higher incidence of chromosomal abnormalities compared to prior years; however, later data indicated a reduction in the percentage of chromosomal abnormalities, reverting to baseline levels (**Figure 4**). Despite the well-documented role of recurrent cytogenetic abnormalities in hematologic malignancies, our analysis did not reveal any significant shifts in the pattern or frequency of these abnormalities in the post-COVID-19 period. All observed abnormalities remained consistent with known recurrent cytogenetic findings typically associated with each respective malignancy, such as t(9;22)(q34;q11) in CML, del(5q) or -7 in MDS, and del(13q) or t(11;14) in MM. Notably, no novel or atypical karyotypic aberrations were identified in any disease group, and the frequency of known abnormalities remained within expected historical ranges.

This suggests that, within the timeframe and sample size of our study, SARS-CoV-2 infection or the broader pandemic context did not exert a detectable influence on the cytogenetic landscape of hematologic cancers. Although transient fluctuations, such as the temporary increase in CLL-related abnormalities in 2023, were observed, these did not persist and may reflect statistical or sampling variations rather than a biological effect.

## Discussion

Although the hypothesis of a possible link between COVID-19 and increased genomic instability is biologically plausible, our study does not provide direct evidence supporting a causal relationship between SARS-CoV-2 exposure and the emergence of hematologic malignancies. Given that cancer is typically a disease with prolonged latency, caution must be taken not to over-interpret short-term fluctuations.

Interestingly, our data revealed a statistically significant reduction in the incidence of AML and myeloproliferative neoplasms (MPN) during the post-COVID-19 period (OR = 0.65 and 0.79, respectively). While these findings may reflect true shifts in referral or diagnosis patterns, they are more likely influenced by external factors such as reduced access to healthcare, diagnostic delays, or changes in patient behavior during the pandemic [10, 11]. Given the chronic nature and often indolent course of these disorders, it is possible that some cases remained undiagnosed or deferred for evaluation until after the study period.

Our findings suggest that the COVID-19 pandemic, including widespread vaccination efforts and associated stressors, did not significantly impact the incidence or cytogenetic profiles of hematologic malignancies over the past 5 years. There were no new recurrent chromosomal abnormalities detected, and no evidence of increased disease occurrence at younger ages. Proportion tests revealed that the frequency of cytogenetic abnormalities remained stable across all disease categories before and after the pandemic, supporting the conclusion that SARS-CoV-2 had no detectable direct cytogenetic effect on hematologic cancers.

Although biological plausibility exists regarding the potential for SARS-CoV-2 to induce genomic instability, our results showed consistent prevalence rates of chromosomal abnormalities in all major disease groups. This indicates that, within our study’s timeframe and sample size, COVID-19 did not appear to significantly influence the cytogenetic landscape of hematologic malignancies. Notably, all chromosomal alterations observed remained within the known spectrum of recurrent abnormalities without emergence of novel karyotypic patterns.

A transient increase in chromosomal abnormalities among CLL cases observed in 2023 was not sustained in subsequent data and may reflect sampling variability rather than a biological effect. This underscores the importance of interpreting single-year fluctuations with caution, particularly in retrospective datasets with variable sample volumes.

Mechanistically, COVID-19 has been associated with disruption of pathways essential for maintaining genomic integrity. SARS-CoV-2 proteins such as NSP7 and NSP13 have been shown to interfere with centrosome function and DNA repair processes [1], potentially elevating the risk of chromosomal instability. Inflammatory responses and oxidative stress induced by COVID-19 may also damage hematopoietic cells [12], particularly in individuals predisposed to malignancy. Additionally, severe infections have been linked to epigenetic reprograming in HSPCs, promoting skewed differentiation and sustained immune dysregulation [5].

Despite these molecular insights, the translation of such cellular-level disruptions into overt cytogenetic changes in clinical disease remains unproven. Several studies have failed to establish a definitive correlation between COVID-19 and the emergence of hematologic cancers [4]. While inflammation and immune dysregulation are recognized contributors to cancer progression, they may not be sufficient to cause the hallmark chromosomal abnormalities typically observed in hematologic malignancies. Rather, these abnormalities are more often driven by inherent genetic susceptibility, environmental exposures, and cumulative genomic insults over time [4].

It is important to emphasize the prolonged latency typically required for cancer development. This makes it unlikely that a viral exposure occurring within the past few years would have already resulted in a measurable increase in *de novo* hematologic malignancies, particularly those requiring clonal evolution and chromosomal rearrangements. Our data reflect this temporal limitation; the absence of increased incidence or novel cytogenetic findings reinforces the view that COVID-19 is not a direct oncogenic trigger, at least in the short term.

Although COVID-19 may exacerbate existing conditions or modulate disease course in patients already diagnosed with hematologic cancers, our study does not support a role for the virus in initiating disease. In fact, some molecular effects attributed to SARS-CoV-2—such as modulation of DNA repair, immune activation, and cytokine signaling—may be insufficient in isolation to induce malignant transformation in hematopoietic cells [13].

In summary, our cytogenetic analysis provides no evidence of an increase in hematologic malignancy incidence or chromosomal abnormalities associated with COVID-19. While biological mechanisms linking the virus to genomic instability are increasingly understood [1], their clinical implications remain theoretical. The absence of significant shifts in karyotypic patterns or disease demographics suggests that hematologic malignancies have remained cytogenetically stable during the pandemic period.

Further studies with longer follow-up periods and broader molecular testing may be required to evaluate potential delayed effects, particularly among high-risk populations or those undergoing chemotherapy. Future investigations incorporating epigenetic and transcriptomic profiling may also provide deeper insights into how COVID-19 interacts with hematopoietic malignancy pathways beyond cytogenetic alterations.

## Conclusion

Our study demonstrates that the incidence, age distribution, and cytogenetic profiles of hematologic malignancies—including leukemia, lymphoma, and bone marrow disorders—remained stable across the 5-year period spanning before and during the COVID-19 pandemic. No new or unexpected recurrent chromosomal abnormalities were identified, and all cytogenetic findings fell within known patterns for each malignancy. Although a transient increase in chromosomal abnormalities among CLL cases was observed in 2023, this fluctuation was not sustained in subsequent data, suggesting variability related to sampling rather than a true biologic effect. These findings indicate that SARS-CoV-2 infection and pandemic-related factors did not exert a measurable influence on the cytogenetic landscape of hematologic malignancies within the analyzed timeframe [14, 15].

While biologically plausible mechanisms exist—such as viral effects on genomic stability, inflammation-induced DNA damage, and epigenetic reprograming—our results do not support a direct association between COVID-19 and increased chromosomal aberrations. The stable cytogenetic findings observed in this cohort align with current evidence indicating that COVID-19 does not function as a short-term oncogenic trigger in hematopoietic cells.

This investigation has limitations. The retrospective nature relied on clinical data collected during a period when healthcare access fluctuated, which may have influenced case numbers and referral patterns. The study also focused solely on cytogenetic abnormalities; therefore, molecular and epigenetic alterations were not evaluated, and the follow-up period may not be sufficient to detect delayed oncogenic effects. As a single-center study, generalizability may be limited.

In conclusion, our findings provide reassurance that hematologic malignancies remained cytogenetically stable during the COVID-19 pandemic. Continued long-term surveillance and incorporation of molecular techniques (e.g., CMA, OGM, NGS) are recommended to evaluate whether subtle genomic or epigenetic effects emerge over time, particularly in vulnerable or predisposed individuals [12, 16].
